# Hints and Observations on Military Hygiene, with the Best Means of Treating the Medical and Surgical Diseases of the Army

**Published:** 1862-06

**Authors:** 


Hints and Observations on Military Hygiene, with the best means of treating the Medical and Surgical Diseases of the Army. By LAURENCE TRUMBALL, M.D., one of the Surgeons of the Howard Hospital. Reprinted from the Medical and Surgical Reporter. Philadelphia. 1862.

This is a monograph of 62 large, double-column, pages, bound in elastic cover; and thereby easily carried by the medical officers of the army, from place to place. From a hasty examination we think the pages well written, and carefully compiled from reliable sources. It is certainly a very useful and convenient manual for the medical officers of the army.

